# High-Resolution, *In Vivo* Magnetic Resonance Imaging of *Drosophila* at 18.8 Tesla

**DOI:** 10.1371/journal.pone.0002817

**Published:** 2008-07-30

**Authors:** Brian Null, Corey W. Liu, Maj Hedehus, Steven Conolly, Ronald W. Davis

**Affiliations:** 1 Stanford Genome Technology Center/Department of Biochemistry/Bio-X Program, Stanford, California, United States of America; 2 Stanford Magnetic Resonance Laboratory, Stanford, California, United States of America; 3 Varian Inc, NMR Instruments, Palo Alto, California, United States of America; 4 Department of Bioengineering, University of California Berkeley, Berkeley, California, United States of America; Baylor College of Medicine, United States of America

## Abstract

High resolution MRI of live *Drosophila* was performed at 18.8 Tesla, with a field of view less than 5 mm, and administration of manganese or gadolinium-based contrast agents. This study demonstrates the feasibility of MR methods for imaging the fruit fly *Drosophila* with an NMR spectrometer, at a resolution relevant for undertaking future studies of the *Drosophila* brain and other organs. The fruit fly has long been a principal model organism for elucidating biology and disease, but without capabilities like those of MRI. This feasibility marks progress toward the development of new *in vivo* research approaches in *Drosophila* without the requirement for light transparency or destructive assays.

## Introduction

The study of tiny, highly tractable model organisms is a powerful paradigm for understanding genetic and biochemical physiology, knowledge readily carried through to vertebrate models and ultimately human medicine. *In vivo* methods for measuring small signaling molecules, metabolites, and neurotransmitters in model organisms, as well as dynamic qualia such as electrical potential, pH, fluid flow and molecular turnover, are highly desirable but very challenging [1]–[5]. To this end Magnetic Resonance (MR), best known for anatomical imaging in widespread clinical applications, is an intriguing method to consider for *Drosophila* research.

To date high field studies of small specimens have demonstrated key achievements bolstering the potential value of the MR techniques for tiny, robust model organisms [6]. Examples include accomplishment of one-micron resolution MR microscopy [7], pH imaging an exceptionally large ant species and studies of other large insects [8], imaging and spectroscopy in single and aggregate *xenopus* oocytes [9], [10], spectroscopy in 200 micron diameter excised *Aplysia californica* neurons [11], spectral editing methods for *in vivo* detection of the neurotransmitter GABA in small rodents [12], honeybee brain imaging [13] and functional studies of the visual system of a very large species of fly [14]. Given these accomplishments, the potential for ultra-high field instrumentation and advanced contrast agents become very intriguing when considered in conjunction with a model organism of great utility like the fruit fly *Drosophila*.

The genetics and molecular biology of the fruit fly are extremely well understood with human and *Drosophila* biology being surprisingly and fortuitously analogous across a broad range of physiological functions. Approximately 75% of known human disease genes have a homolog in the *Drosophila* genome, and 50% of fruit fly protein sequences have mammalian analogs [15], [16]. Accordingly much of our current molecular understanding of human biology is rooted in and enabled by *Drosophila* research. In fact a wide variety of human disorders including developmental, metabolic, and neurodegenerative diseases, tumorigenesis, and many others, are studied in *Drosophila* for insights to their molecular pathology and treatment [17]–[19]. With a strong molecular characterization of the genome, transcriptome, proteome, and developmental cascade [20]–[25], the fly is arguably the most thoroughly understood and tractable of any organism of morphological complexity greater than that of a worm, *C. elegans*. At the least, *Drosophila* constitutes a rich system to elucidate complex molecular physiology with a level of control that is impossible in humans or mammal models. This study is a feasibility demonstration for application of ultra-high field MR to *Drosophila*, accomplished with an existing spectrometer instrument similar to others sited at Nuclear Magnetic Resonance (NMR) facilities worldwide.

## Results

**Figure 1:**
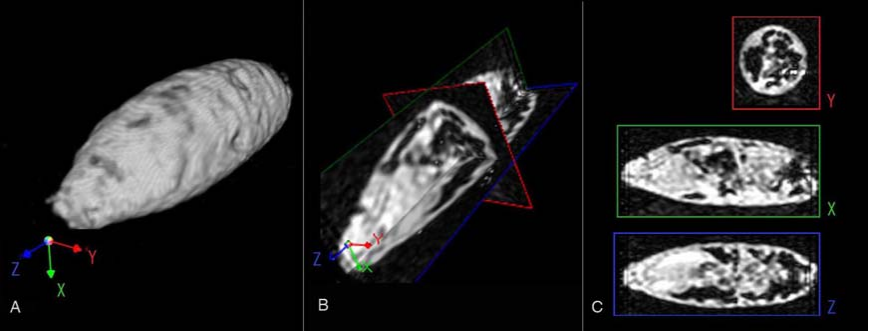
Early *D. melanogaster* pupa. A) Exterior view B) Triple cross-section C)Three separated cross-sectional slices. Sequence: Spin Echo 3D, gadopentate dimeglumine administered in food medium during larval stages, imaged in air, 10°C, resolution 19.5 microns, matrix: 128×128×256 TR: 195 ms, TE: 9.6 ms, FOV 2.5×2.5×5 mm (Data also provided as supplementary material file “Movie S1”).

**Figure 2:**
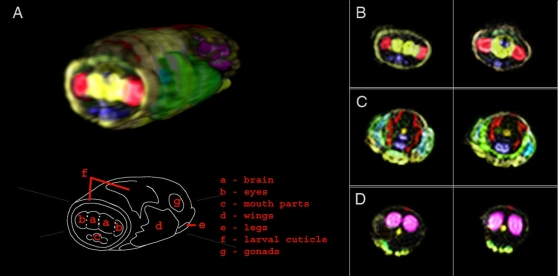
Late *D. melanogaster* pupa. A) Exterior view and labelled schematic. At right, selected example slices from the B) head C) thorax and D) abdomen. Sequence: Spin Echo Multi-Slice, 41 slices, 100 micron thickness, resolution: 12.5 micron, in-plane. Matrix: 128×128, TR = 11300 ms, TE = 20 ms.

*Drosophila* adults and pupae from a range of developmental stages were imaged at high resolution by several distinct modes of imaging. Figures 1 and 2 show *Drosophila* pupae in the earliest and latest stages of metamorphosis. At the developmental stage depicted in figure 1, the larva has begun the pupal transition by anchoring itself to the glass surface of the container with glue proteins secreted from the salivary glands, which appear here with high relative signal along the ventral interior of the head. Attachment of the larva to a surface and formation of an immotile cocoon through hardening of the cuticle marks the initiation of metamorphosis, a massive developmental reorganization in which the larval body will be digested and re-formed to the adult morphology. Adult tissues present as ‘imaginal discs’ tethered to the larval brain by nervous tissue will grow and develop, taking approximately four days at 25°C to reach the next major developmental transition point, depicted in figure 2. This late-stage pupa was imaged just prior to emergence of the adult fly from the cocoon. The eyes (highlighted red) can be seen on either side of, and densely connected, to the two hemispheres of the brain, below which are the mouthparts (blue). The wings are folded on either side of the body (green), the legs packed tightly to the body in rows along the ventral side, and the putative gonads are highlighted in the abdomen (magenta). A feature corresponding to the putative dorsal vessel, which acts as the heart pump of the fly's open circulatory system, is marked as a thin red line along the posterior dorsal region of the abdomen.

**Figure 3:**
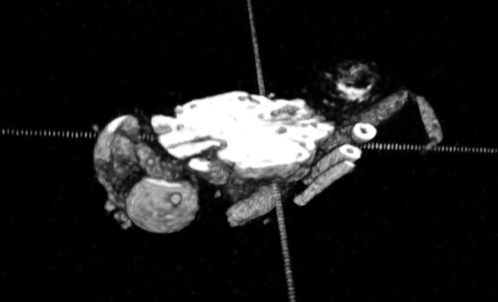
Adult *D. melanogaster*, dorsal slices omitted. Sequence: Gradient Echo 3D; resolution: 19.5 microns, matrix: 128×128×256, field of view (FOV): 2.5×2.5×5 mm. TR: 195 ms, TE: 9.6 ms. Injected with gadopentate dimeglumine contrast agent during pupal stage, imaged in halocarbon oil.

**Figure 4:**
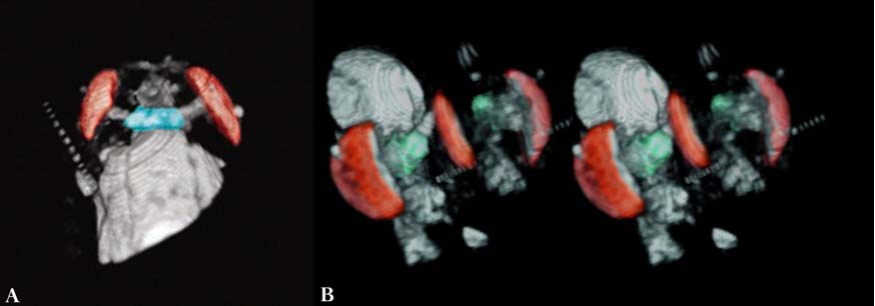
Adult *D. melanogaster*, head and anterior thorax of specimen in figure 3. A) Dorsal view B) Anterior view, stereograph. The brain is highlighted blue-green, surfaces of the eyes red. Partial transparency of the head cuticle was achieved by adjusting per cent image density after 3D rendering.

Figures 3 and 4 show an adult *Drosophila*, imaged whole by three-dimensional gradient-echo pulse sequence, and virtually sectioned midway through the dorso-ventral axis of the thorax. In figure 4 the head and anterior thorax region of the fly is shown, in dorsal view and an anterior oblique view stereograph, in which a portion of the head has been virtually dissected and lifted away to show internal structures. Color-highlighted are the brain and surface of the eyes, with clear definition of the optical stalk bundles connecting them, which contain the neuronal processes transmitting visual information to the brain. The cuticle, eyes, and mouthparts can be readily discerned, and some musculature and other internal structures are also discernible in this data set.

**Figure 5:**
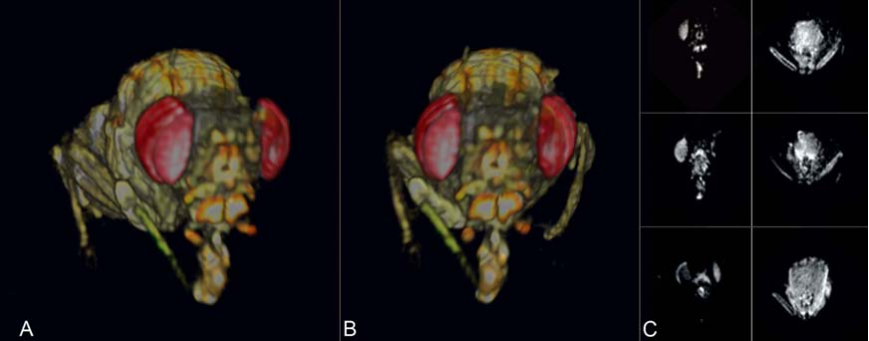
Adult *D. bifurca*. A,B) Two exterior views of 3D rendering. C) Example 2D slices from the image set, three from the head, left, and three from the thorax, right. Sequence: Spin Echo Multi-Slice, 12.5 micron in-plane resolution, 100 micron slice thickness, TR = 1500 ms, TE = 20 ms, matrix: 128×128, FOV: 1.6×1.6 mm.

A spin echo imaging sequence with moderate T_2_ contrast was used to generate figure 5, an adult *D. bifurca*. The multi-slice 2D spin echo sequence required a much longer total acquisition time than with the use of a 3D method and contrast agent as in figure one, although we note that a 3D spin echo sequence might provide reasonable compromise between exogenous contrast and total imaging time. We observed anecdotally that specimens prepared with contrast agent administration yielded useable global signal strength with the relaxation time (TR) reduced to as little as one-tenth of that used without contrast agent, dramatically reducing the total acquisition time and thus improving temporal resolution.

## Discussion

The likely role of MR microscopy is not to supersede the utility of light microscopy, but to add intriguing capabilities beyond and complementary to those of light microscopy. In terms of spatial and temporal resolution, light microscopy is plainly superior to MR for imaging transparent and dissected or vivisected specimens. Furthermore the application of high resolution light microscopic methods to biology is ubiquitous, with new techniques such as ultra thin tissue sectioning and *in vivo* two-photon microscopy pushing new bounds in this area [26], [27]. However MR does not require light transparency and is also non-deleterious, fitting an alternative role for full-volume, *in vivo* imaging of individual specimens, with spectral capabilities not possible by light microscopy.

*Drosophila* pupae are particularly apt subjects for MR analyses, undergoing development of the adult body within an opaque cocoon; impossible to image live *in toto* by conventional light microscopy. Pre-pupae naturally affix themselves to glass or plastic surfaces in culture, and are viable in perfluorocarbon oil immersion, used to reduce magnetic susceptibility effects and improving magnetic field homogeneity. The pupal stages are ripe for biological study and, as we have demonstrated, well suited to MR preparation.

MR contrast agents are analogous to optical dye molecules of conventional microscopy. An agent like gadopentate dimeglumine alters relaxivity of resonating nuclei, thereby improving signal with shorter relaxation times (TR). The relaxivities of magnetic resonance contrast agents and the T_1_ relaxation time values of tissues are strongly field dependent, with relaxation times being the dominant portion of total acquisition time; thus contrast agents at high field improve definition of a tissue with much shorter acquisition time. Conventional clinical contrast agents like these are used to improve signal-to-noise ratio generally, and in some cases highlight specific tissues or lesions. More exciting are recent advances in contrast agents that include a calcium ion concentration indicator, a UAS/Gal4 gene expression reporter, and an expressible protein contrast agent, analogous in potential utility to the Green Fluorescent Protein type of reporters pervasive in molecular/cellular techniques using light microscopy [5], [6].

On the issue of potential applications of MR techniques to *Drosophila*, there are some conceivable directions to pursue. For example, the ability to image and quantify a neurotransmitter such as GABA, and couple this ability with existing techniques such as high-throughput (microarray) gene expression data, mutant studies, and RNA interference techniques, would yield a new totality of information with potential for improving and rapidly integrating human disease models. Previously, many molecules like GABA have been found to be difficult or impossible to detect amongst the complex milieu of chemical resonance signatures *in vivo*, but development of spectral editing methods show that GABA, and other previously undetectable molecules, are quantifiable in living cells [12]. Another speculative possibility to consider is the use of MR contrast agent-labelled insecticide compounds since insecticidal compounds have been intensely studied and bind to known ion channels in cell membranes. The use of these compounds as targeted labels of fly homologues to human receptors might comprise an intriguing tool for research, particularly when coupled with the fly model's existing strength in genomic and molecular approaches. Embryonic-stage flies (eggs) provide a rich area for further research due to being famously well studied by other methods, and are also viable in halocarbon oil. Embryos present a more challenging starting point for MR microscopy than *Drosophila* pupae or adults due to their very small dimensions. However, at about 500×200 µm, fly embryos approach the size range of cells imaged in at least one prior study [11], indicating that embryos may yet be feasible candidates for MR imaging and spectroscopy. Notably, the fly embryo undergoes a pre-cellularized, multinucleate (syncitial) stage of development, which is extremely advantageous for transfection techniques introducing artificial constructs into cells of the fly. While there have been advances, the utility of MR contrast agent indicators of cellular physiology and gene expression has been limited by the administration of contrast agents to the interior of cells. It remains to be seen whether the fly's syncitial development can be used to similar advantage in overcoming this bottleneck.

## Materials and Methods

These studies were performed on a Varian 800 MHz NMR instrument with vertical bore magnet (18.8 T, Oxford Instruments), INOVA console and ^1^H/^13^C/^15^N liquids spectroscopy probe with triple-axis gradients for 5 mm diameter sample tubes (Varian, Inc.). Except as noted, specimens were immersed in gas-permeable fluorocarbon oil, a high molecular weight polymer of chlorotrifluoroethylene, (Halocarbon, Inc.). This oil was used to prevent dessication and improve matching of magnetic susceptibility between the specimen and the surrounding medium. Pupal flies survived to eclosion (adulthood) in this oil following imaging. Imaging sequences utilized were as provided in the standard VNMR spectrometer software package from Varian Inc., and include spin-echo multi-slice, 3D spin echo, and 3D gradient echo [28]. All sequences utilized conventional (spin warp) phase encoding in one (2D sequences) or two (3D sequences) dimensions. 3D renderings and movies were created using the ‘Volocity’ software package (Improvision Inc.). A frame-by-frame ‘fly-through’ of the early stage pupa in Figure 1 is provided in supplementary materials as ‘Movie S1’. Larger, false-color reconstruction movies are viewable via the communicating author's website, or by direct communication.

Contrast Agents: Either gadopentate dimeglumine contrast agent (‘Magnevist’, Berlex Inc.) or a solution of 100 mM Manganese Chloride were administered, either by direct feeding or microinjection, during larval, pupal, or adult phases of the life cycle. Detailed notes and findings regarding specimen preparation, materials and methods are open-archived via the *Drosophila* Information Service: http://www.ou.edu/journals/dis/DIS90/Technique/Null.pdf

## Supporting Information

Movie S1 Early Pupa, magnevist labeled, Spin Echo 3D; X,Y,&Z axis fly-through.
(1.21 MB AVI)
